# Supplementary material for: SiSaNA: a Python-based command line interface for single-sample network analysis
Source: Bioinformatics. 2026 Oct 30;42(11):btag798. doi: 10.1093/bioinformatics/btag798 (PMC13750893; doi:10.1093/bioinformatics/btag798)
Supplement: Supplementary file 1 [file btag798_supplementary_data.zip › btag798_Supplementary_Data.html]

Python Results


# SiSaNA results

## Preprocessing

After removing **93** genes that were not present in at least 5 samples, **4908** genes were retained for the network reconstruction step.

## Volcano plot

To interpret this plot, pay attention to the TFs/genes that are colored. These are the genes that are below the **0.25** FDR threshold and greater (absolute value) than the threshold of **50** set for the difference in median degree. These may be genes that are important in distinguishing your two groups from one another.

There are **823** genes higher in LumB and **580** genes higher in LumA.

## Quantity plot

Below you will find the heatmap created for visualizing the genes. Column clustering was not performed. Row clustering was performed.

## GSEA results

Below you will find the top pathways that are enriched between the two sample groups. The GMT file used for generating these results was c2.cp.reactome.v2023.2.Hs.symbols.gmt.
